# Diversity and frequency of *kdr* mutations within *Anopheles sinensis* populations from Guangxi, China

**DOI:** 10.1186/s12936-016-1467-3

**Published:** 2016-08-15

**Authors:** Chan Yang, Xiangyang Feng, Zushi Huang, Mei Li, Xinghui Qiu

**Affiliations:** 1 State Key Laboratory of Integrated Management of Pest Insects and Rodents, Institute of Zoology, Chinese Academy of Sciences, Beijing, 100101 China; 2 Guangxi Zhuang Autonomous Region Centre for Diseases Control and Prevention, Nanning, 530028 China; 3 University of Chinese Academy of Sciences, Beijing, 100049 China

**Keywords:** *Anopheles sinensis*, Voltage-gated sodium channel (*VGSC*), Knockdown resistance (*kdr*), Haplotype, Genealogical analysis, Guangxi Zhuang Autonomous Region

## Abstract

**Background:**

*Anopheles sinensis* is a major vector of malaria in China and its control is under great threat as the development of insecticide resistance. Voltage-gated sodium channel (*VGSC*) is the target of several classes of insecticides. Genetic mutations of *VGSC* have been documented to confer knockdown resistance (*kdr*) to dichlorodiphenyltrichloroethane (DDT) and pyrethroids in mosquitoes. To control this vector efficiently, it is important to know the resistance-associated genetic mutations, their distribution frequencies and genealogical relations.

**Methods:**

Three hundreds and thirteen (313) adults of *An. sinensis* collected from nine locations across Guangxi Zhuang Autonomous Region were used. The partial sequence of the *An. sinensis* voltage gated sodium channel gene (*AS*-*VGSC*) containing codon 1014 was sequenced. PHASE2.1 was used to construct the haplotypes of each individual, and the accuracy of haplotypes was further confirmed by clone sequencing. The genealogical relations of *kdr* mutations in *AS*-*VGSC* was analysed using TCS 2.1 and Network 5.0.

**Results:**

Sixteen *AS*-*VGSC* haplotypes including seven haplotypes carrying non-synonymous mutations at codon 1014, and fifty-five *AS*-*VGSC* genotypes were identified from 313 mosquitoes collected from nine geographical locations across Guangxi. The number of haplotypes in each of the nine populations ranged from 5 to 13. The frequency of haplotypes carrying *kdr* mutations ranged from 2.7 to 80.0 % within the nine populations, of which 1014C was unexpectedly high in the northeast of Guangxi. Genealogical analysis suggested multiple origins of *kdr* mutations in *An. sinensis*.

**Conclusion:**

Diverse haplotypes of *AS*-*VGSC* are distributed in Guangxi. The presence of haplotypes carrying mutations at codon 1014 indicates a risk of pyrethroid and DDT resistance. The *kdr* mutations show differential distribution geographically, with high frequencies occurred in the northeast of Guangxi. Genealogical analysis suggests multiple origins of *kdr* mutations in *An. sinensis* populations in Guangxi. These findings have important practical implications for the sustainability of *An. sinensis* control programmes.

**Electronic supplementary material:**

The online version of this article (doi:10.1186/s12936-016-1467-3) contains supplementary material, which is available to authorized users.

## Background

*Anopheles sinensis* is a major vector of malaria in China and countries of Southeast Asia. Chemical control of vector has played an important role in malaria control and elimination [1]. In China, DDT has been widely used for indoor residual spray (IRS) since 1950s, and pyrethroids for insecticide-treated bed nets (ITNs) since 1980s [2]. Unfortunately, long-term and large-scale use of pyrethroids has led to increasing insecticide resistance in Chinese *An. sinensis* [3, 4], which poses a major threat to malaria control.

The voltage-gated sodium channel protein is the major target for pyrethroids and DDT [5]. Although there have been debates [6–8], many studies have demonstrated that mutations at codon 1014 of *VGSC* cause resistance to both pyrethroids and DDT in many arthropod species [5, 6]. 1014F/S/H mutations can reduce sodium channel sensitivity to pyrethroids in *Xenopus* oocytes [9–15], and provide protection to pyrethroids and DDT [9]. In *An. sinensis*, significant positive correlations between *kdr* allele frequency and bioassay-based resistance phenotype have been documented [16–19]. Recent years, *kdr* mutation has been used as a molecular mark for monitoring pyrethroid resistance in *An. sinensis* in China [19].

Guangxi Zhuang Autonomous Region was once a malaria-endemic region. Before 1949, there were more than 5 million malaria patients per year [20]. Thanks to the “National Malaria Control Programme”, the “Basically Eliminating Malaria” strategy and the “Action Plan of Malaria Elimination (2010–2020)” implemented since 1955, 2000, and 2010 respectively in China [21, 22], the malaria burden has been substantially reduced, with one indigenous and 2068 imported cases being reported from 2010 to 2015 in Guangxi [22]. Although Guangxi has already achieved remarkable accomplishments in eliminating malaria, the risk of malaria re-emergence remains partly due to increasing cross-border population migration and the unique natural environment (e.g. rice fields) suitable for mosquito breeding in Guangxi [22–25].

As the historical use in vector control and continuing use in agriculture of different insecticides, not only the geographical distribution and density of malaria vectors are likely to change, insecticide resistance is expected to be selected as well. Recent investigations have shown that *An. sinensis* has replaced *Anopheles minimus* to be the main malaria vector in Guangxi [24]. However, the status of insecticide resistance in *An. sinensis* is less understood in this region up to date. As an effort in this direction, the distribution and frequency of *VGSC* mutations that possibly lead to resistance to DDT and pyrethroids were investigated in nine field populations of *An. sinensis* collected extensively across Guangxi in this study.

## Methods

### Samples

*Anopheles sinensis* adults used in the study were caught by light trap (wave length 365 nm) from July to September in 2014, around farmers’ houses at different geographical locations across Guangxi. The sampling sites were part of the malaria vector monitoring sites that were set up partly because there were imported malaria cases observed since 2010 in these regions. A brief description of the sampling sites was provided in Additional file 1. The farmers living at the sampling sites usually use mosquito-coil (largely containing pyrethroids) to prevent mosquitoes, and apply insecticides (e.g. pyrethroids) for crop protection.

The collected individual mosquitoes were first morphologically identified using the determination key provided by [26]. *Anopheles sinensis* adults were put into 100-μl Eppendorf tubes containing 100 % ethanol solution, and kept at 4 °C until use. The accuracy of morphological classification was guaranteed by molecular detection of ten randomly selected adults from each population using the rDNA-ITS2 method [27].

### PCR amplification of partial *AS*-*VGSC* sequence

The genomic DNA of individual adult mosquitoes was prepared as described elsewhere [28, 29]. A fragment containing codon 1014 of *AS*-*VGSC* gene was amplified using primers kdr-F (5′-TGCCACTCCGTGTGTTTAGA-3′) and *kdr*-R (5′-GAGCGATGATGATCCGAAAT-3′) [16]. The PCR mixture (30 μl) consisted of 6 μl DNA template, 0.4 μl Takara Taq Polymerase, 3 μl 10xTaq Buffer, 3 μl 2.5 μM dNTPs, 0.6 μl kdr-F, 0.6 μl kdr-R and 16.4 μl ddH_2_O. The reaction programme was 95 °C for 5 min, followed by 36 cycles each with 95 °C for 30 s, 55 °C for 30 s, 72 °C for 30 s, and by a final extension of 10 min at 72 °C. PCR products were gel-purified and sequenced using the forward primer *kdr*-F (BGI, China).

### Haplotype identification and genealogical analysis

Genotype data were carefully inspected by using Chromas (Technelysium Pty Ltd, Australia). *AS*-*VGSC* haplotypes were constructed by software PHASE2.1 (http://www.bioinf.man.ac.uk/resources/phase/) from the genotype data. The presence (and accuracy) of haplotypes constructed in heterozygotes was confirmed by sequencing of three to five clones derived from 12 representative heterozygotes. Genealogical relations among haplotypes were analysed using Network 5.0 [30] and TCS2.1 [31].

## Results

### Identification of polymorphic sites in the *AS*-*VGSC* gene

**Fig. 1:**
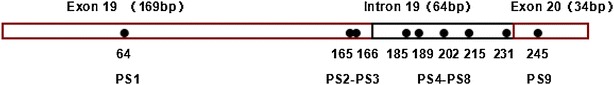
The polymorphic sites (PS) identified in the fragment of AS-VGSC gene

The gene of *An. sinensis* voltage sensitive sodium channel (KE525266.1)contains 31 exons and 30 introns. A ~325 bp-in-length sequence was sequenced through direct sequencing of each purified PCR product. The partial sequence (267 bp), excluding the primer sequence and covering partial exon 19 (169 bp), intron 19 (64 bp)and partial exon 20 (34 bp), was used for further analysis (Fig. 1). Two synonymous mutations (C/G at PS1, T/C at PS9) were observed. Five polymorphic sites were found in the intron 19 (PS4–8). Non-synonymous mutations were identified in the second (T/G/C) and third nucleotide (G/T) of codon 1014 (PS2 and 3).

### Haplotype diversity of *AS*-*VGSC* gene

From the sequence data of 313 individuals, sixteen *AS*-*VGSC* haplotypes were identified (Table 1). The number of haplotypes in each of the nine populations ranged from 5 (Guigang) to 13 (Yulin). Three haplotypes (1014L2, 1014L3 and 1014L8) were detected in all the nine populations, while 1014L7 was uniquely distributed in Nanning. 1014L1 was the predominant haplotype for Nanning and Yulin, 1014L2 for Baise and Hechi, and 1014L8 was most abundant in Wuzhou (Table 2).

**Table 1:** Haplotypes identified or/and used in this study

Haplotype	Polymorphic sites	Intron type	Known distribution in China (Province)^a^
1014L1	CTG*ACGTT*C	H1	Fujian, Guangxi, Guangdong, Guizhou, Hainan, Henan, Sichuan, Yunnan
1014L2	CTG*ACGCC*T	H2	Fujian, Guangxi, Guangdong, Guizhou, Hainan, Henan, Sichuan, Yunnan
1014L3	CTG*ACTCC*T	H3	Fujian, Guangxi, Guizhou, Hainan, Henan, Sichuan, Yunnan
1014L4	CTG*ACTTC*T	H4	Fujian, Guangxi, Hainan, Yunan
1014L5	GTG*ACGCC*T	H2	Fujian, Guangxi, Guangdong, Hainan, Sichuan
1014L6	CTG*TCGCC*T	H5	Guangxi, Hainan, Sichuan, Yunnan
1014L7	CTG*ACGTC*C	H6	Guangxi, Yunnan
1014L8	CTG*ATGCC*T	H7	Guangxi, Hainan
1014L9	CTG*ACTTC*C	H4	Guangxi
1014L10^b^	CTG*ACGTC*T	H6	Guizhou, Shandong
1014L11^b^	CTG*ACTTT*C	H8	Hainan
1014L12^b^	CTG*ACTCC*C	H3	Sichuan
1014L13^b^	CTG*TCGCC*C	H5	Yunnan
1014L14^b^	CTG*ACGCT*T	H9	Anhui
1014F1	CTT*ACGTT*C	H1	Anhui, Guangxi, Guizhou,Henan, Hubei, Jiangsu, Shandong
1014F2	CTT*ACTCC*T	H3	Anhui, Guangxi, Hainan, Henan, Hubei, Jiangsu
1014F3^b^	CTC*ACTCC*T	H3	Hubei, Jiangsu, Shandong
1014F4^b^	CTT*ACTTT*C	H8	Anhui
1014F5^b^	CTC*ACGTC*C	H6	Henan
1014F6^b^	CTT*ACGCC*T	H2	Henan
1014S1	GCG*ACGCC*T	H2	Anhui, Guangxi, Guangdong, Henan
1014S2	CCG*ACGCC*T	H2	Guangxi, Guangdong
1014S3	CCG*ACTCC*T	H3	Guangxi
1014S4	CCG*ACTTC*T	H4	Guangxi
1014C1	CGT*ACGTT*C	H1	Anhui, Guangxi, Guizhou, Hubei, Jiangsu
1014C2^b^	CGT*ACTTT*C	H8	Anhui

^a^Distribution information is adopted from cited references and this study. The polymorphic sites within intron 19 are in italics

^b^Mean that these haplotypes are retrieved from GenBank (1014L10 = KP763768, 1014L11 = KP763787, 1014L12 = KP763792, 1014L13 = KF697673, 1014L14 = KF697679, 1014F3 = KP763782, 1014F4 = KF697680, 1014F5 = KF927156, 1014F6 = KP763803, 1014C2 = KF697683)

**Table 2:** Haplotypes of the *AS*-*VGSC* gene and their frequencies (%) in nine *An. sinensis* populations from Guangxi, China

Haplotype	BS	GG	GL	HC	HZ	LZ	NN	WZ	YL
1014L1	27.3		10.5	33.3		13	*38.6*	10.4	*31.7*
1014L2	*51.8*	6.7	13.2	*38.5*	10.5	11.1	24.3	16.7	14.4
1014L3	9.1	3.3	10.5	3.1	2.6	9.3	17.1	4.2	8.7
1014L4	0.9		2.6			3.7	5.7		1.9
1014L5	1.8		4.0			3.7	1.4	8.3	1.9
1014L6	0.9		1.3	1.0			4.3		4.8
1014L7							1.4		
1014L8	4.6	10.0	7.9	12.5	7.9	3.7	2.9	*27.1*	22.1
1014L9	0.9								1.0
1014F1		6.7	13.2	1.0	7.9	16.7		2.1	2.9
1014F2			1.3		2.7	1.8			
1014S1				1.1			1.4	8.3	
1014S2	0.9			2.1			2.9		2.9
1014S3	0.9			5.2					1.0
1014S4	0.9			1.1		1.8		2.1	3.8
1014C1		*73.3*	*35.5*	1.1	*68.4*	*35.2*		20.8	2.9
Mutant	2.7	80.0	50.0	11.6	79.0	55.5	4.3	33.3	13.5
Size (2 N)	110	30	76	96	38	54	70	48	104

Mutant means 1014C1, 1014F1, 1014F2, 1014S1, 1014S2, 1014S3, 1014S4 in this study. The italics value represents the frequency of the most abundant haplotype in the corresponding population. BS, GG, GL, HC, HZ, LZ, NN, WZ and YL are the abbreviations of Baise, Guigang, Guilin, Hechi, Hezhou, Liuzhou, Nanning, Wuzhou and Yulin respectively

**Fig. 2:**
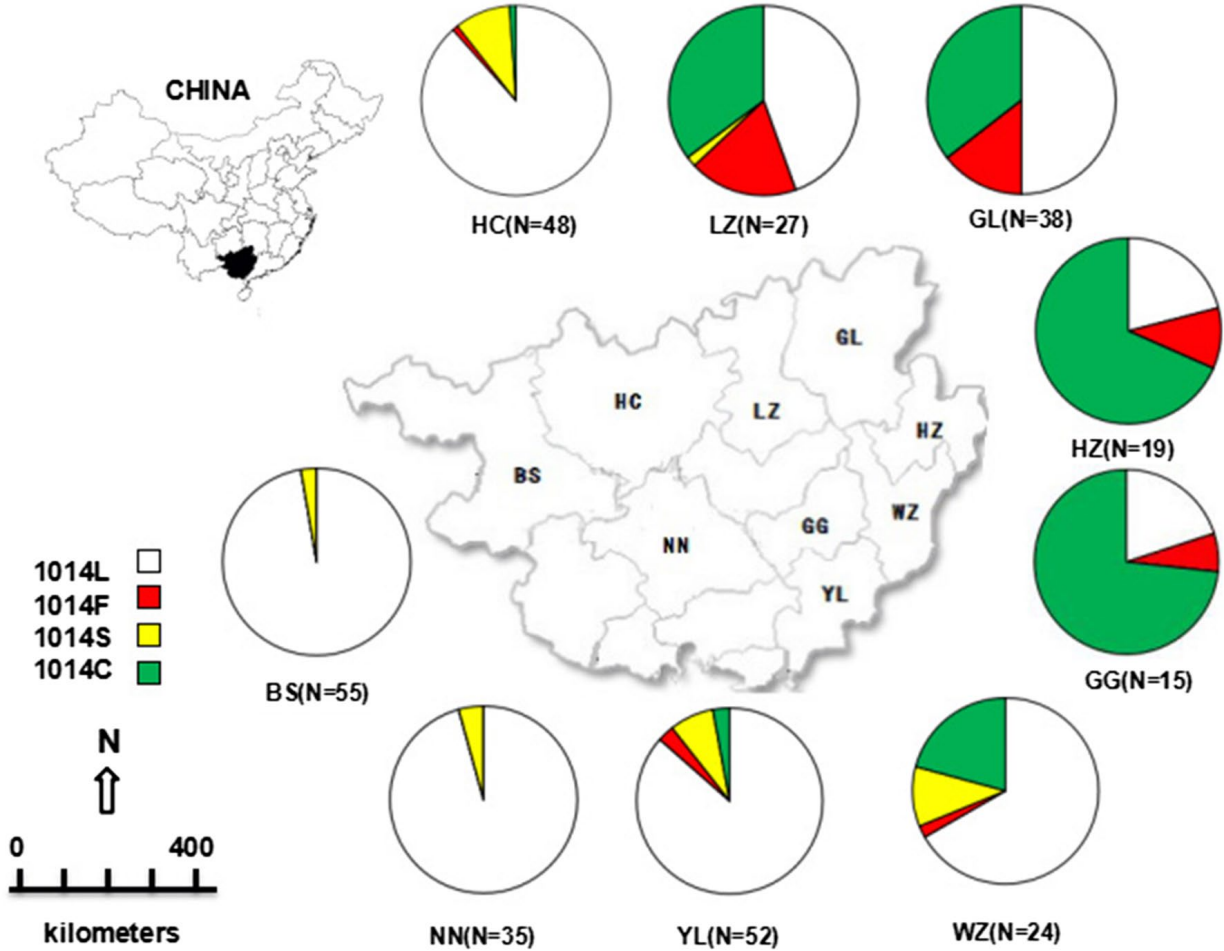
Distribution of haplotypes in *Anopheles sinensis* in Guangxi. BS, HC, HZ, GG, GL, LZ, NN, WZ and YL are the abbreviations for Baise, Hechi, Hezhou, Guigang, Guilin, Liuzhou, Nanning, Wuzhou and Yulin respectively

Point mutations at position 1014 of the domain II of the *VGSC* protein have been documented to confer *kdr* [5, 6]. In this study, three types of *kdr* mutations were detected: TTT (1014F), TCG (1014S), and TGT (1014C). Accordingly, seven haplotypes carrying *kdr* mutations, namely 1014F1, 1014F2, 1014S1, 1014S2, 1014S3, 1014S4 and 1014C1 respectively were identified (Table 1), in the frequencies ranging from 2.7 to 80.0 % in the nine populations (Fig. 2). In the four populations in the northeast (Guigang, Guilin, Hezhou and Liuzhou), the frequencies of *kdr* mutations were more than 50.0 %, predominated by 1014C1. In contrast, only 1014S in a low frequency (<5 %) was observed in the two western populations (Baise and Nanning). Three types of *kdr* mutations coexisted in the other three populations (Hechi, Wuzhou, Yilin) in a frequency from 11.6 to 33.3 %.

Fifty-five *AS*-*VGSC* genotypes were detected (Additional file 2). Among the nine populations, the predominant genotype was largely population-specific. According to the encoding amino acid at position 1014, all the 55 genotypes could be grouped into wild-type (1014L/1014L), mutant-type (1014F/1014F, 1014C/1014C and 1014F/1014C), heterozygous-type (1014L/1014S, 1014L/1014C and 1014L/1014F) (Table 3). Wild-type was found in all the nine populations, in frequencies ranging from 5.3 to 94.6 %. No mutant-type individual was observed in Baise, Hechi, Nanning and Yulin. The mutant homozygote 1014F/1014F was only observed in Hezhou in a low frequency of 3.7 %, while 1014C/1014C homozygote was detected in Guigang (60.0 %), Hezhou (42.1 %), Liuzhou (22.3 %), Guilin (15.8 %) and Wuzhou (8.3 %).

**Table 3:** Frequency of *VGSC* genotypes in nine *An. sinensis* populations from Guangxi China, grouped according to the amino acid at position 1014 of *VGSC*

Genotype	BS	GG	GL	HC	HZ	LZ	NN	WZ	YL
1014 L/L	*94.6*	6.7	*29.1*	*77.0*	5.3	18.5	*91.3*	*41.6*	*73.4*
1014 L/F		6.7	15.7	2.1		*25.9*		4.2	5.7
1014 L/S	5.4			18.8		3.7	8.7	20.9	15.2
1014 L/C		20.0	26.3	2.1	31.6	22.2		25.0	5.7
1014 F/F						3.7			
1014 C/C		*60.0*	15.8		*42.1*	22.3		8.3	
1014 F/C		6.7	13.1		21.0	3.7			
Size (N)	55	15	38	48	19	27	35	24	52

The italics value represents the frequency of the most abundant genotype in the corresponding population. BS, GG, GL, HC, HZ, LZ, NN, WZ and YL are the abbreviations of Baise, Guigang, Guilin, Hechi, Hezhou, Liuzhou, Nanning, Wuzhou and Yulin

### Genealogical analysis of *kdr* mutations

**Fig. 3:**
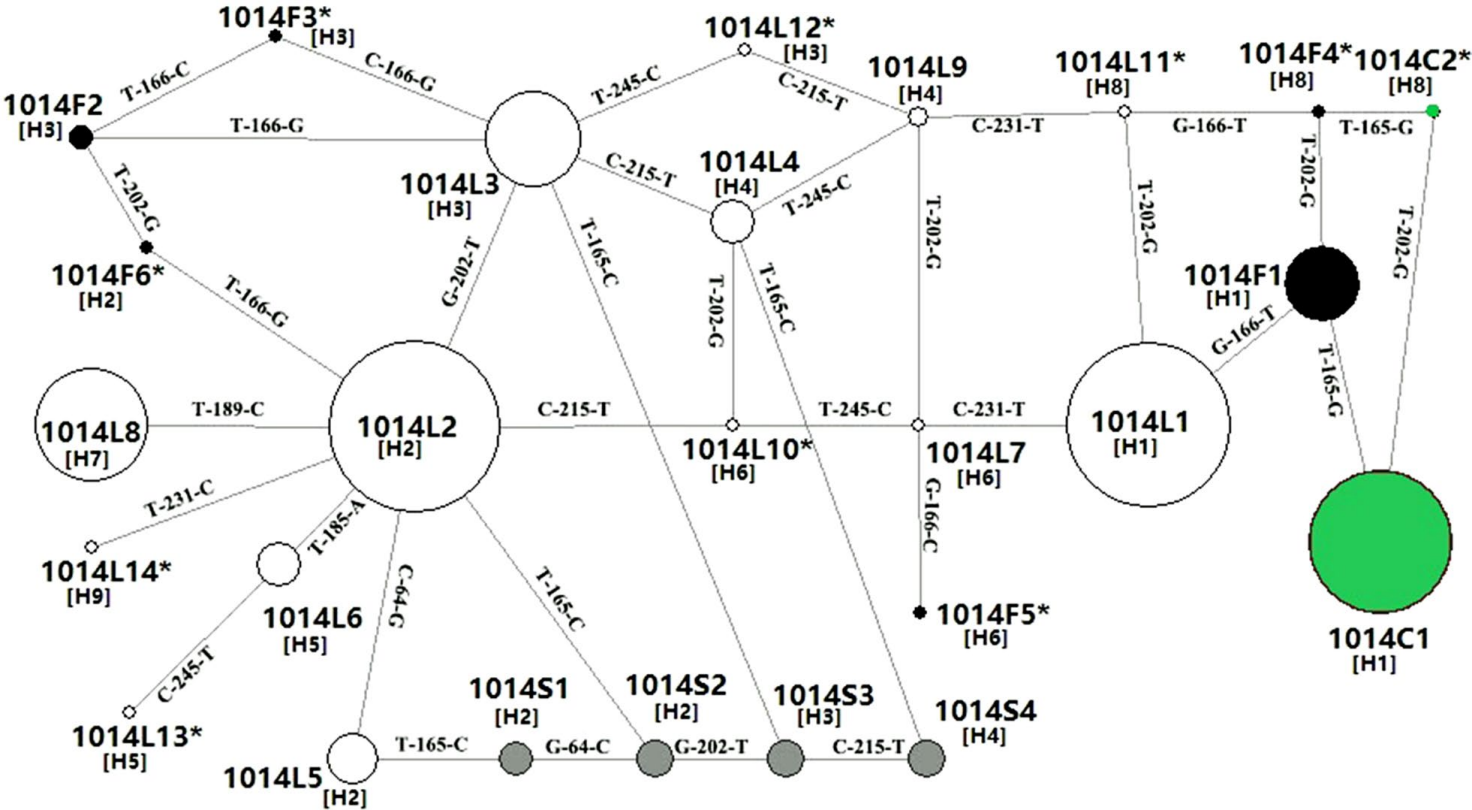
Network showing the genealogy of *AS*-*VGSC* haplotypes. *Empty circle* means the wildtype haplotype (without 1014 mutation), *solid circles* mean mutant haplotypes with 1014 mutation (*black* 1014F, *grey* 1014S, *dark green* 1014C). The size of each *circle* is proportional to the corresponding frequency of a certain haplotype identified in Guangxi province. *Straight line* indicates the possible mutational step. The *note* above the *line* referred to the mutation position and base. Haplotypes marked with *Asterisk* mean that these haplotypes are retrieved from GenBank. *H* represents the type of intron haplotypes (see Table 1 for details)

To estimate the evolutionary relationship of *kdr* mutations, sixteen haplotypes identified in this study and ten haplotypes retrieved from GenBank database (Table 1) were used to construct a network using TCS 2.1 and Network 5.0. The two software generated identical results for our dataset, thus only the network produced by Network 5.0 was displayed (Fig. 3).

The network analysis revealed complex reticulate pattern and multiple independent mutation events leading to *kdr* haplotypes. For example, haplotype 1014F1 was possibly derived from 1014L1, while 1014F2 from 1014L3; 1014S1, S2, S3 and S4 were perhaps the results of an independent mutational step from four different wild haplotypes 1014L5, 1014L2, 1014L3 and 1014L4 respectively.

The genealogical analysis also revealed that a single mutation (T165C) might result in the resistant phenotype from the susceptible one. For example, 1014S2 shared identical intron sequence with 1014L2, L5 and S1, and a single mutation (T165C) might change the susceptible 1014L2 to the resistant 1014S2.

## Discussion

Sequence analysis reveals that the sodium channel gene of *An. sinensis* has diverse genetic mutations. From 313 mosquitoes collected from Guangxi, 16 haplotypes were identified (Tables 1, 2). The number and frequency of *AS*-*VGSC* haplotypes are different among the nine populations from Guangxi. Overall, the 1014L1, 1014L2 and 1014C1 rank the top three haplotypes. Some haplotypes, for example 1014L2, 1014L3 and 1014L8, are widespread, and some haplotypes such as 1014L7 are restricted to a particular location (Table 2). Less number of haplotypes detected in Hezhou and Guigang may be related to the relative small sample size and/or strong selective pressure leading to 1014C1 in a high frequency. The high polymorphism of *AS*-*VGSC* may be partly explained by their large population size, wide distribution range of *An. sinensis* [14], diverse natural landscapes and different local insecticide selective pressure.

Three non-synonymous mutations (TTT for 1014F, TGT for 1014C and TCG for 1014S) were identified in this work. However, the TTC-encoding 1014F, previously found in Korea [32] and in Anhui and Sichuan of China [19], was not detected in Guangxi. The presence of L1014W substitution was reported based on the direct sequencing data of only one *An. sinensis* heterozygote from Guiping of Guangxi [17]. This result is questionable and needs reconfirmation, because 1014F/W is not the only possibility encoded by the individual genotype T(T/G)(T/G). In this and another study [33], sequencing of cloned fragment with the T(T/G)(T/G) template resolved into two alleles, TTG and TGT, encoding 1014L/C rather than 1014F/W.

Differential *kdr* distribution patterns were observed in the nine examined *An. sinensis* populations from Guangxi. Overall, haplotypes carrying 1014C mutation are widespread and present in high frequencies in the northeast, while 1014S is rare and distributed in the west (Table 1; Fig. 2). Notably, four of the nine populations with *kdr* allele frequencies higher than 50 % were located in the northeast of Guangxi, while only 1014S in a frequency less than 5 % was detected in the two western populations. Geographically, the frequency of *kdr* mutations decreased towards south and west from northeast. This pattern is in keeping with previous observations that a high *kdr* mutation frequency was detected in the central China which is located northeast to Guangxi [19, 34, 40], and *kdr* alleles were less prevalent in Yunnan and Hainan which are in the west and south to Guangxi respectively [16, 18, 19, 34, 40].

Considering the fact that haplotype 1014C1 and 1014F1, detected in samples of this study, are also distributed in central China (Table 1), it is possible that migration of *An. sinensis* population from central China through Hunan and Guizhou, may contribute to the occurrence of 1014C1 and 1014F1 in the northeast of Guangxi. The obvious distinction in the geographic distribution of each allele between populations in the west and in the northeast is likely a combined consequence of independent mutational events in different geographic locations and the geographical barriers limiting gene flow imposed by the mountainous landscapes of Guangxi. Theoretically, selective pressure may play a role in shaping the frequency of insecticide resistance-associated mutation in a population. However, it was not possible to assess the contribution of local selection of insecticides to the existing *kdr* distribution pattern because application history of insecticides was not known for samples used in this study.

Previous studies have documented that the predominant *kdr* allele is L1014 F [4, 12, 13, 16, 19, 33–35, 40], the high frequency of 1014C1 in the five *An. sinensis* populations in northeast of Guangxi (Fig. 3) is unexpected. 1014C was present in *An. sinensis* samples collected in Anhui, Guizhou, Henan, Hubei, Hunan, Jiangsu and Shandong of China [16, 19, 34, 40] and in Korea [33], and was also found in *VGSC* of other mosquito species, such as *Culex pipiens pipiens* from China [36] and *Anopheles albimanus* from Mexico and Nicaragu [37]. These observations indicate that 1014C is a conserved mutation in mosquitoes and widely distributed. Why 1014C1 is prevalent in the five populations remains to be investigated. One possibility is that 1014C1 may provide a better protection, or/and a lower fitness cost, than other *kdr* alleles such as the classical 1014F.

The geographic distribution pattern and the genealogical analysis of *kdr* haplotypes strongly suggests that *kdr* mutations are not singly originated. For example, there are at least two independent mutation events giving rise to 1014F or 1014S haplotypes from a wild haplotype through a single mutational step (Table 1; Fig. 3). Multiple origins of resistance alleles via point mutations at the voltage-gated sodium channel gene have also been characterized in other insect species [38, 39]. Interestingly, 1014C1 and 1014F1 is co-distributed in Guangxi and share the same intron (Tables 1, 2). The network analysis indicates that only one mutational step is able to change 1014F1 to 1014C1 (Fig. 3). Based on these observations, it is hypothesized that 1014C1 may represent a *kdr* allele evolved via a sequential progression from 1014F1 and perhaps has gradually replaced 1014F1.

## Conclusions

Sixteen *AS*-*VGSC* haplotypes were identified in *An. sinensis* from Guangxi, suggesting diverse genetic mutations in the *AS*-*VGSC* gene. Three types of *kdr* mutations (1014F, 1014S and 1014C) were detected in Guangxi. Genealogical analysis suggests multiple origins of *kdr* mutations in *An. sinensis.* Although a direct link between *kdr* mutations and level of insecticide resistance has not been established due to the unavailability of susceptibility data for the samples used in this study, the high frequency of *kdr* mutation (>50 %) in Guigang, Guilin, Hezhou and Liuzhou indicates a risk of resistance to pyrethroids and DDT in *An. sinensis* in these areas. The heterogeneities in the geographic distribution and multiple origins of *kdr* alleles highlight the need of a location-customized strategy for monitoring and management of insecticide resistance.

## Additional files

10.1186/s12936-016-1467-3 Brief description of *Anopheles sinensis* collection sites in Guangxi.
10.1186/s12936-016-1467-3 Genotypes of the *AS-VGSC* gene and their frequency in nine *An. sinensis* populations from Guangxi, China.

## Abbreviations

*VGSC*: voltage-gated sodium channel
*kdr*: knockdown resistance
DDT: dichlorodiphenyltrichloroethane
ITNs: insecticide-treated bed nets
IRS: indoor residual spray
