# Supplementary material for: Diversity and frequency of kdr mutations within Anopheles sinensis populations from Guangxi, China
Source: Malar J. 2016 Aug 15;15:411. doi: 10.1186/s12936-016-1467-3 (PMC4986192; doi:10.1186/s12936-016-1467-3)
Supplement: Supplementary file 1 — 10.1186/s12936-016-1467-3 Brief description of Anopheles sinensis collection sites in Guangxi. [file 12936_2016_1467_MOESM1_ESM.doc]

**Table S-1. Brief description of *Anopheles sinensis* collection sites in Guangxi**

| Population | Geographic location | Landscape/environment |
| --- | --- | --- |
| Baise  (BS) | 105°34′E, 24°77′N  106°42′E, 23°13′N  106°34′E, 24°21′N | Mountain forest region, rice fields |
| Guigang  (GG) | 110°43′E, 23°30′N | Plain, rice fields |
| Guilin  (GL) | 110°19′E, 25°25′N  110°02′E, 25°80′N  109°99′E, 24°98′N | Karst mountainous region, rice fields |
| Hechi  (HC) | 107°58′E, 23°44′N  108°26′E, 24°83′N  107°22′E, 24°31′N  107°10′E, 25°00′N | Karst Landform, rice and sugarcane fields |
| Hezhou  (HZ) | 111°28′E, 24°81′N | Karst Landform, hills, rice fields |
| Liuzhou  (LZ) | 109°36′E, 25°47′N | Karst Landform, hills , rice and vegetable fields |
| Nanning  (NN) | 108°48′E, 23°13′N | Plain, Rice and vegetable fields |
| Wuzhou  (WZ) | 111°16′E, 23°28′N | Hills, rice and vegetable fields, and rivers |
| Yulin  (YL) | 110°15′E, 22°18′N 110°36′E, 22°71′N | Plain, rice and vegetable fields |
