# Supplementary material for: Diversity and frequency of kdr mutations within Anopheles sinensis populations from Guangxi, China
Source: Malar J. 2016 Aug 15;15:411. doi: 10.1186/s12936-016-1467-3 (PMC4986192; doi:10.1186/s12936-016-1467-3)
Supplement: Supplementary file 2 — 10.1186/s12936-016-1467-3 Genotypes of the AS-VGSC gene and their frequency in nine An. sinensis populations from Guangxi, China. [file 12936_2016_1467_MOESM2_ESM.doc]

**Table S-2. Genotypes of the *AS-VGSC* gene and their frequency in nine *An. sinensis* populations from Guangxi, China.**

| Genotype | BS | GG | GL | HC | HZ | LZ | NN | WZ | YL |
| --- | --- | --- | --- | --- | --- | --- | --- | --- | --- |
| 1014L1/1014L1 | 16.4 |  | 2.6 | 8.3 |  |  | 11.4 |  | 7.7 |
| 1014L1/1014L7 |  |  |  |  |  |  | **2.8** |  |  |
| 1014L1/1014L2 | 16.4 |  | 2.6 | 22.9 |  | 7.4 | 14.3 | 8.3 | 9.6 |
| 1014L1/1014L8 | 1.8 |  | 5.3 | 14.6 |  |  | 2.8 | 4.1 | 13.5 |
| 1014L1/1014L6 | 1.8 |  |  |  |  |  | 2.8 |  |  |
| 1014L1/1014L5 |  |  |  |  |  |  | **2.8** |  |  |
| 1014L2/1014L2 | 30.9 |  |  | 18.7 | 5.3 | 3.7 | 8.6 | 4.1 | 1.9 |
| 1014L2/1014L8 | 5.5 | 6.7 |  | 6.2 |  |  |  | 8.3 | 5.8 |
| 1014L2/1014L6 |  |  |  |  |  |  | **2.8** |  |  |
| 1014L2/1014L5 | **1.8** |  |  |  |  |  |  |  |  |
| 1014L8/1014L8 |  |  |  |  |  |  |  | 4.1 | 5.8 |
| 1014L6/1014L8 |  |  |  |  |  |  |  |  | **3.9** |
| 1014L5/1014L8 |  |  | 2.6 |  |  |  |  | 4.1 | 3.9 |
| 1014L1/1014L4 | 1.8 |  |  |  |  |  | 5.7 |  | 3.9 |
| 1014L2/1014L4 |  |  | 2.6 |  |  |  | 2.8 |  |  |
| 1014L4/1014L8 |  |  |  |  |  |  | **2.8** |  |  |
| 1014L4/1014L5 |  |  | 2.6 |  |  | 3.7 |  |  |  |
| 1014L2/1014L9 | 1.8 |  |  |  |  |  |  |  | 1.9 |
| 1014L3/1014L3 | 1.8 |  | 2.6 |  |  |  | 2.8 |  |  |
| 1014L1/1014L3 |  |  | 2.6 | 2.1 |  | 3.7 | 20.0 |  | 7.7 |
| 1014L2/1014L3 | 12.8 |  | 2.6 | 4.2 |  |  | 5.7 | 4.1 | 3.9 |
| 1014L3/1014L8 |  |  |  |  |  |  |  | **4.1** |  |
| 1014L3/1014L6 |  |  | 2.6 |  |  |  | 2.8 |  | 3.9 |
| 1014L3/1014L5 | **1.8** |  |  |  |  |  |  |  |  |
| 1014F1/1014F1 |  |  |  |  |  | **3.7** |  |  |  |
| 1014C1/1014C1 |  | 60.0 | 15.8 |  | 42.1 | 22.3 |  | 8.3 |  |
| 1014L1/1014S4 |  |  |  | 2.1 |  | 3.7 |  |  | 3.9 |
| 1014L2/1014S4 | 1.8 |  |  |  |  |  |  |  | 1.9 |
| 1013L3/1014S4 |  |  |  |  |  |  |  |  | **1.9** |
| 1014L1/1014S1 |  |  |  |  |  |  |  | **4.1** |  |
| 1014L8/1014S1 |  |  |  |  |  |  |  | **8.3** |  |
| 1014L1/1014S3 |  |  |  | **6.2** |  |  |  |  |  |
| 1014L2/1014S3 |  |  |  | 2.1 |  |  |  |  | 1.9 |
| 1014L8/1014S3 | 1.8 |  |  | 2.1 |  |  |  |  |  |
| 1014L1/1014S2 |  |  |  |  |  |  | 2.8 |  | 1.9 |
| 1014L2/1014S2 | 1.8 |  |  | 2.1 |  |  | 2.8 |  |  |
| 1014L8/1014S2 |  |  |  | 2.1 |  |  |  |  | 1.9 |
| 1014L6/1014S2 |  |  |  |  |  |  |  |  | **1.9** |
| 1014L5/1014S4 |  |  |  |  |  |  |  | **4.1** |  |
| 1014L2/1014S1 |  |  |  | 2.1 |  |  | 2.8 | 4.1 |  |
| 1014L6/1014F1 |  |  |  | **2.1** |  |  |  |  |  |
| 1014L4/1014F1 |  |  |  |  |  | **3.7** |  |  |  |
| 1014L3/1014F2 |  |  |  |  |  | **3.7** |  |  |  |
| 1014L3/1014F1 |  |  |  |  |  | **7.4** |  |  |  |
| 1014L2/1014F1 |  | 6.7 | 7.9 |  |  |  |  |  |  |
| 1014L8/1014F1 |  |  | 2.6 |  |  | 3.7 |  | 4.1 | 1.9 |
| 1014L5/1014F1 |  |  | **2.6** |  |  |  |  |  |  |
| 1014L1/1014F1 |  |  | 2.6 |  |  | 7.4 |  |  | 3.9 |
| 1014L1/1014C1 |  |  | 2.6 | 2.1 |  | 3.7 |  | 4.1 | 3.9 |
| 1014L2/1014C1 |  |  | 10.5 |  | 10.5 | 7.4 |  |  |  |
| 1014L8/1014C1 |  | 13.3 | 5.3 |  | 15.8 | 3.7 |  | 12.5 | 1.9 |
| 1014L3/1014C1 |  | 6.7 | 7.9 |  | 5.3 | 3.7 |  |  |  |
| 1014L5/1014C1 |  |  |  |  |  | 3.7 |  | 8.3 |  |
| 1014F2/1014C1 |  |  | 2.6 |  | 5.2 |  |  |  |  |
| 1014F1/1014C1 | 15.8 | 6.7 | 10.5 |  |  | 3.7 |  |  |  |
| Size (N) | 55 | 15 | 38 | 48 | 19 | 27 | 35 | 24 | 52 |

**Note: The underlined value represents the frequency of the most abundant genotype and the highlighted represent the unique genotype in the corresponding population. BS, GG, GL, HC, HZ, LZ, NN, WZ and YL are the abbreviations of Baise, Guigang, Guilin, Hechi, Hezhou, Liuzhou, Nanning, Wuzhou and Yulin.**
